# Alterations of Coherent Theta and Gamma Network Oscillations as an Early Biomarker of Temporal Lobe Epilepsy and Alzheimer’s Disease

**DOI:** 10.3389/fnint.2018.00036

**Published:** 2018-08-27

**Authors:** Valentina F. Kitchigina

**Affiliations:** Institute of Theoretical and Experimental Biophysics, Russian Academy of Sciences (RAS), Pushchino, Russia

**Keywords:** Alzheimer’s disease, temporal lobe epilepsy, memory, electroencephalography, oscillatory activity, coherent analysis, early diagnostics

## Abstract

Alzheimer’s disease (AD) and temporal lobe epilepsy (TLE) are the most common forms of neurodegenerative disorders characterized by the loss of cells and progressive irreversible alteration of cognitive functions, such as attention and memory. AD may be an important cause of epilepsy in the elderly. Early diagnosis of diseases is very important for their successful treatment. Many efforts have been done for defining new biomarkers of these diseases. Significant advances have been made in the searching of some AD and TLE reliable biomarkers, including cerebrospinal fluid and plasma measurements and glucose positron emission tomography. However, there is a great need for the biomarkers that would reflect changes of brain activity within few milliseconds to obtain information about cognitive disturbances. Successful early detection of AD and TLE requires specific biomarkers capable of distinguishing individuals with the progressing disease from ones with other pathologies that affect cognition. In this article, we review recent evidence suggesting that magnetoencephalographic recordings and coherent analysis coupled with behavioral evaluation can be a promising approach to an early detection of AD and TLE.

**Highlights**

– Data reviewed include the results of clinical and experimental studies.
– Theta and gamma rhythms are disturbed in epilepsy and AD.
– Common and different behavioral and oscillatory features of pathologies are compared.
– Coherent analysis can be useful for an early diagnostics of diseases.

## Introduction

It is known that theta and gamma oscillations are closely related to cognitive processes. The theta rhythm (4–12 Hz) is prominent oscillations recorded in the hippocampus and surrounding limbic structures during exploration and REM sleep (Vanderwolf, 1969; Bland, 1986; Buzsáki, 2002). Theta oscillations have also been registered in the neocortex where they are particularly pronounced in the frontal midline (Klimesch et al., 1997; Kahana et al., 1999; Jensen and Tesche, 2002; Canolty et al., 2006; Guderian et al., 2009), and in the subcortical areas (Paré et al., 2002; Magill et al., 2006; Nerad and McNaughton, 2006; DeCoteau et al., 2007; Kabanova et al., 2011). The theta rhythm is important in the formation and retrieval of episodic and spatial memory (Hasselmo, 2005). The gamma rhythm (25–100 Hz) usually co-occurs with theta rhythm in the hippocampus (Bragin et al., 1995; Strogatz, 2003; Montgomery et al., 2008). In the neocortex, gamma oscillations were identified in the frontal and parietal areas (Bouyer et al., 1981; Benchenane et al., 2010). The gamma rhythm is considered to play a role in attention (Fries, 2009; Jutras et al., 2009; Buzsáki and Wang, 2010) and in the maintenance of relevant information in memory (Sauseng et al., 2009; Sridharan and Knudsen, 2015). Evidence accumulates indicating that the coupling between the phase of slow oscillations (in particular, theta) and the amplitude of fast oscillations (gamma) may be involved in information processing (Tort et al., 2009; Canolty and Knight, 2010; Lisman and Jensen, 2013).

A crucial component of the neural processing underlying cognition is communication between selective brain structures (Livanov et al., 1977; Engel et al., 2001; Vinogradova, 2001; Igarashi et al., 2014). Mounting evidence points to brain rhythms as a fundamental mechanism of dynamical coupling between brain areas; this is proved by task- and state-dependent changes in the coherence of local field potentials (LFPs; Fell et al., 2001; Varela et al., 2001; Buzsáki, 2004; Womelsdorf et al., 2006; Sauseng et al., 2008; Takehara-Nishiuchi and McNaughton, 2008; Astasheva et al., 2016; Bott et al., 2016; Vinck et al., 2016) and cross-correlated unit activity (Tabuchi et al., 2000; Engel et al., 2001; Igarashi et al., 2014). Synchronized activities of brain areas exert strong effects on their ability to interact with each other (Womelsdorf et al., 2006), and provide a mechanism for the formation of cell ensembles and their coordination by linking the activity of multiple neurons (Harris et al., 2003; Colgin and Moser, 2010; Buzsáki, 2010; Buzsáki and Watson, 2012; Igarashi et al., 2014). Besides, the oscillations can be considered as rhythmic changes in neuronal excitability (Volgushev et al., 1998; Fries, 2005).

The hypothesis “communication through coherence” by Fries (2005) is now widely accepted (Jensen et al., 2007; Mitchell et al., 2008; Colgin et al., 2009; Rutishauser et al., 2010; Wang, 2010; Colgin, 2011, 2015; Igarashi et al., 2014; Astasheva et al., 2016). This hypothesis assumes that anatomic communications can become effective or inefficient owing to presence or lack of rhythmic synchronization (Fries, 2005; Bastos et al., 2015).

It is known that communication between selective brain structures as well as oscillatory activity in them can violate in neurological and psychiatric disorders (Bakker et al., 2012; Buzsáki and Watson, 2012; Froriep et al., 2012; Kirihara et al., 2012; Inostroza et al., 2013; Laurent et al., 2015). However, there is much to be learned and discussed. Despite decades of research, the disturbances in the rhythm coherence underlying pathologies, such as temporal lobe epilepsy (TLE) and Alzheimer’s disease (AD) remain poorly understood.

This review article summarizes the data on the alterations of the theta and gamma coherence based on examples from TLE and AD. In addition, we analyzed the information on some similarities and differences in these disorders, mainly in the disturbances of specific types of memory, theta and gamma rhythms and their coherence. These analyses may shed light on plausible links between neural damage and rhythmic disturbances in these diseases and help to design new approaches to early diagnostics of pathologies.

## Coherence of the Theta and Gamma Rhythms

**Figure 1:**
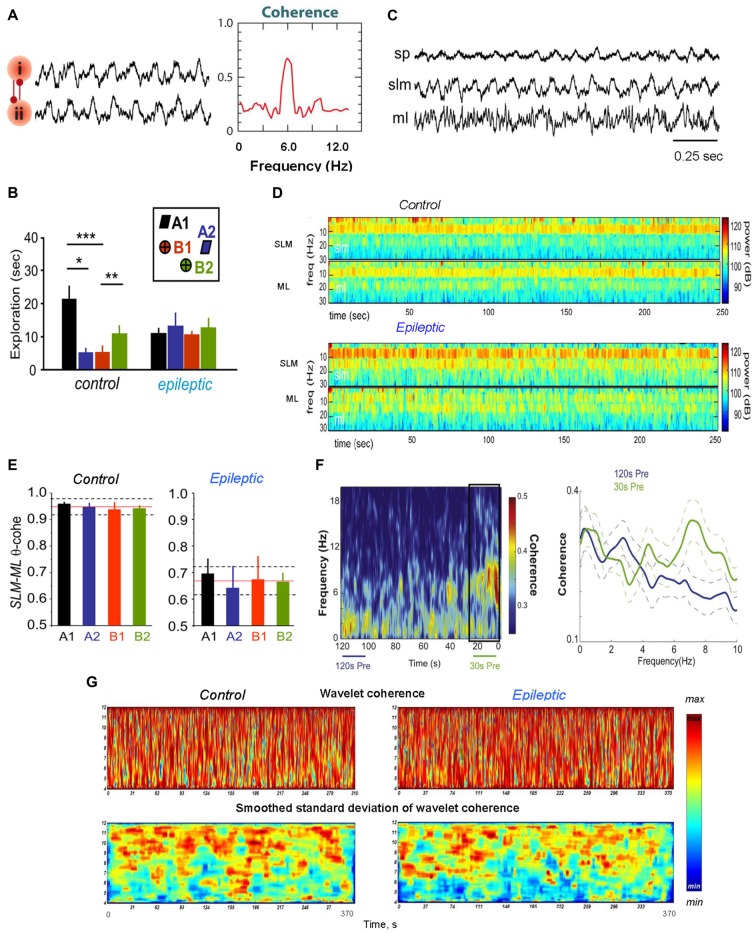
Theta coherence between brain areas changes during epileptogenesis. **(A)** Phase–phase coupling of theta oscillations between two brain areas (i) and (ii). To the left: synthetic data used for theta rhythm illustration. To the right: coherence spectrum (or phase-specific measures) between two signals can determine the strength of theta phase coupling. **(B)** Behavioral data for rats during the performance the episodic-like memory task. Distribution of exploratory times per object in the test phase for the control and epileptic groups; *p < 0.05, **p < 0.01, ***p < 0.005. The inset represents the object configuration in the task. **(C)** Representative hippocampal activity of an epileptic rat recorded in the stratum pyramidale (SP), lacunosum moleculare (SLM) and moleculare (ML) during walking. **(D)** Specific alterations in hippocampal theta activity in temporal lobe epilepsy (TLE) brain during object exploration in the episodic-like memory task; the time–frequency power spectrum of hippocampal field potentials in the SLM and the ML layers is shown for the 1–30 Hz frequency band. **(E)** Theta coherence between hippocampal SLM–ML layers during exploration of each individual object in the episodic-like memory task; the mean values of theta coherence per object within the mean (red line) and standard deviation (discontinuous line) for the whole session in the control and epileptic animals are shown. **(F)** Theta coherence between the hippocampus and medial prefrontal cortex (mPFC) increases pre-ictally. To the left: a mean coherogram (coherence vs. time, 0–20 Hz) of 120 s and 30 s pre-ictal local field potential (LFP) segments from the hippocampus and mPFC (30 s pre-ictal segment is designated by a black rectangle). To the right: mean ± standard error of the mean (SEM; solid ± dashed lines) coherence of 120 s (blue lines) and 30 s (green lines) recordings before seizures. **(G)** Representative wavelet coherograms and smoothed standard deviation of wavelet coherence of LFPs recorded in the hippocampus and medial septal-diagonal band (MSDB) in healthy (left) and epileptic animals. Adapted with permission from Buzsáki and Watson (2012) **(A)**, Inostroza et al. (2013) **(B–E)**, Broggini et al. (2016) **(F)** and Kabanova et al. (2011) **(G)**.

The brain cortex generates great number of oscillations at different frequencies. Low-frequency brain rhythms are dynamically involved across distributed brain regions by sensory signals or cognitive tasks; at the same time, high-frequency brain activity reflects local cortical processing (Canolty and Knight, 2010). External or internal events can lead to the synchronization of rhythms and thus form a more complex functional phenomenon known as phase coherence or phase coupling (Fell et al., 2008; Cavanagh et al., 2009; Canolty and Knight, 2010). The standard phase coherence reveals the relative constancy of the phase difference between two oscillations of the same frequency, i.e., within-frequency synchrony (Rodriguez et al., 1999; Hurtado et al., 2004). It was shown that phase coupling reflects various cognitive processes in humans (Canolty et al., 2006; Axmacher et al., 2010), monkeys (Canolty and Knight, 2010), rats (Montgomery and Buzsáki, 2007; Tort et al., 2008, 2009; Nácher et al., 2013) and mice (Wulff et al., 2009). The within-frequency phase coupling between oscillations in different brain areas (see Figure 1) was studied extensively because of its proposed role in the regulation of inter-area communications (Womelsdorf et al., 2006; Gregoriou et al., 2009; Siegel et al., 2009). Similarly to within-frequency synchrony, the cross-frequency phase–phase coupling, may serve as a mechanism for regulation of communications between different spatiotemporal scales (Palva et al., 2005, 2010; Holz et al., 2010). Besides, the correlation between the amplitude envelopes of two brain waves at different frequencies, called cross-frequency amplitude–amplitude coupling, is also an oscillatory characteristic (Shirvalkar et al., 2010; Tanninen et al., 2017). The amplitude–amplitude cross-frequency coupling was observed by some authors (Friston, 1997; Palva et al., 2010; Shirvalkar et al., 2010), but despite correlations with behavior, its functional role remains unclear.

**Figure 2:**
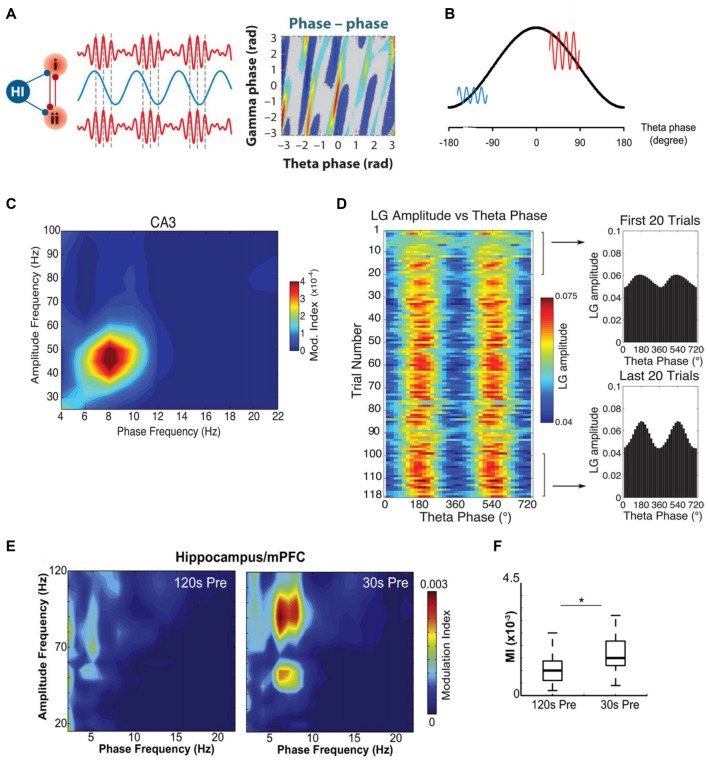
Theta–gamma cross-frequency coupling (CFC) and its alteration in a rat model of TLE. **(A)** Schematic illustration of cross-frequency phase-phase coupling. Phases of theta and gamma oscillations are correlated, as shown (to the right) by the phase-phase plot of the two frequencies; (i) and (ii)—different brain areas, Hi—hippocampus. **(B)** A heuristic model of cross-frequency phase–amplitude coupling. Gamma oscillations are large (red line) in the excitatory phase of theta wave (black line) and small (blue line) in the inhibitory phase of theta wave. **(C)** The theta phase modulates the low-frequency gamma (LG) amplitude. A phase–amplitude comodulogram computed for LFP of the hippocampal CA3 field recorded at SP during execution of spatial task is shown. **(D)** Theta modulation of the LG amplitude in the CA3 region during context exploration increases with learning. Color scale representation of the mean LG amplitude as a function of the theta phase for each trial in the session (left). The mean LG amplitude per theta phase averaged over the first and last 20 trials is also shown (right). **(E)** Example of comodulation maps of hippocampal theta phase modulating mPFC gamma oscillation amplitude 120 s and 30 s before seizure onset. **(F)** Box plot showing mean hippocampal theta/mPFC gamma modulation index (MI), 120 s and 30 s before seizure onset; *p < 0.001. Adapted with permission from Buzsáki and Watson (2012) **(A)**, Kirihara et al. (2012) **(B)**, Tort et al. (2009) **(C,D)** and Broggini et al. (2016) **(E,F)**.

The phase coupling between theta and gamma oscillations, namely, the phase–amplitude cross-frequency coupling (phase–amplitude CFC) or “nested” oscillations (Buzsáki et al., 1983, 2003; Soltesz and Deschênes, 1993; Bragin et al., 1995; Lisman and Idiart, 1995; Mormann et al., 2005; Canolty et al., 2006; Sirota et al., 2008; Tort et al., 2008, 2009; Tort et al., 2010; Sauseng et al., 2009; Wulff et al., 2009; Scheffer-Teixeira et al., 2012; Schomburg et al., 2014) and the phase–phase CFC (or “n:m phase-locking”) in which several gamma cycles are entrained within one cycle of theta (Tass et al., 1998; Belluscio et al., 2012; Zheng and Zhang, 2013; Xu et al., 2015; Zheng et al., 2016) are the most studied phenomena of phase coherence. The phase–amplitude CFC describes the dependence between the phase of the low-frequency rhythm and the amplitude of the high-frequency oscillations (Canolty and Knight, 2010; see Figure 2). Thus, it reflects the interrelations between local microscale (Colgin et al., 2009; Quilichini et al., 2010) and system-level macroscale neuronal networks (Lisman and Idiart, 1995; Canolty and Knight, 2010; Szczepanski et al., 2014). This is probably the most prominent “law” underlying the hierarchy of the system of brain oscillators, when the phase of slower oscillations modulates the amplitude of a faster rhythm (or rhythms; Bragin et al., 1995; Buzsáki, 2006; Buzsáki and Mizuseki, 2014). Thus, phase–amplitude CFC can be used as an index of cortical excitability and network interactions (Knight, 2007; Haider and McCormick, 2009; Voytek et al., 2013). In non-epileptogenic hippocampi of neurosurgical patients and in a healthy brain of rodents, the degree of theta–gamma phase–amplitude coupling increases with learning (Tort et al., 2008, 2009; Lega et al., 2016). In the hippocampus, gamma and theta oscillations normally show a marked phase–amplitude CFC considered to be central to hippocampal functions (Tort et al., 2008, 2009; Newman et al., 2013). Thus, during spatial learning, the strength of hippocampal theta–gamma coupling usually directly correlated with the increase in correct performance of a cognitive task (Tort et al., 2009; Figure 2C).

At the same time, phase–phase CFC provides, as believed, a physiological mechanism for the linkage of the activity generated at significantly different rates. Since gamma oscillations are faster than theta ones, numerous cycles of gamma arise during a single cycle of theta (Figures 2A,B). The phenomenon of phase–phase theta–gamma coupling means that gamma waves always begin at the same phase of theta waves. Phase–phase CFC was hypothesized to take part in cognitive processes, such as attention and memory (Lisman and Idiart, 1995; Schack and Weiss, 2005; Sauseng et al., 2009; Holz et al., 2010; Fell and Axmacher, 2011). An influential model in which theta and gamma oscillations would interact to produce a neural code (“7 ± 2 short-term memories”) has been proposed a decade ago (Lisman and Idiart, 1995); later it was improved, but the essence of this theta–gamma coding model was remained (Jensen and Lisman, 2005; Lisman, 2005; Lisman and Buzsáki, 2008; Lisman and Jensen, 2013). Latest findings show that this mechanism indeed is used by the hippocampus (Belluscio et al., 2012; Zheng and Zhang, 2013; Xu et al., 2015; Zheng et al., 2016; but see Scheffer-Teixeira and Tort, 2016). It is assumed that the temporal coordination of neuronal spikes by phase–phase theta–gamma coupling may improve transferring information as well as spike timing-dependent plasticity (Markram et al., 1997; Fell and Axmacher, 2011). Desynchronization of these rhythms could be altered in certain neurodegenerative pathologies.

## Coherence Bias in TLE and AD

### Alterations in Rhythm Coherence in the Epileptic Brain

Epilepsy, a disorder associated with increased network excitability and neuron loss, is usually accompanied by rewiring in the brain (for review see Morimoto et al., 2004). TLE is the most common and pharmacologically resistant type of adult focal epilepsy. In patients with TLE, a selective and marked degradation of episodic (autobiographic) memory was shown, in which specific memory items are placed within temporal context during encoding and retrieval (Dupont et al., 2000). Animals with TLE also exhibited a highly specific impairment of the episodic-like memory while preserving other forms of hippocampal-dependent memories (Burgess et al., 2002; Helmstaedter, 2002; Tulving, 2002; Chauvière et al., 2009).

#### Hippocampal Network

The analysis of hippocampal LFPs in neurosurgical patients during the execution of episodic memory tasks revealed a sharp increase of gamma oscillations before successful item encoding in non-epileptogenic hippocampi. At the same time, the epileptogenic hippocampi exhibited a significant decrease in the gamma band power, which predicts successful item encoding (Lega et al., 2012, 2015). Thus, typical changes in the gamma band power during this process are reversed for human epileptogenic hippocampus (Lega et al., 2015). Besides, it was shown in the TLE model (Inostroza et al., 2013) that kainate-treated rats with deficit of episodic-like memory exhibited reduction of hippocampal theta power and coherence along the CA1–dentate axis. In TLE animals, decreased theta coherence in the LFP signals was concentrated between the hippocampal stratum lacunosum-moleculare (SLM) and ML of dentate gyrus (DG; Figures 1D,E). Inostroza et al. (2013) believe that these data point to discoordination of hippocampal inputs from layers III and II of the entorhinal cortex (EC) and from the contralateral hippocampus as a possible cause for dysfunction of episodic-like memory in TLE animals.

#### Hippocampal–Entorhinal Cortex Network

It is known that a crucial mechanism of episodic memory is the coherence of neuronal activity in the hippocampal–entorhinal circuit; this mechanism is usually impaired in TLE (Helmstaedter, 2002). An alteration of theta coherence between the EC and the DG was revealed in behaving kainate-injected epileptic mice during the interictal phase (Froriep et al., 2012). Indeed, in epileptic mice, the theta activity in the EC was delayed with respect to that of the DG, while the theta activity in healthy animals was synchronized between EC and DG, demonstrating the within-frequency phase coupling. On the basis of a computational neural mass model, the authors suggested that hippocampal cell loss destroyed the coupling of the subnetworks, which induced the EC–DG shift (Froriep et al., 2012).

In experiments with healthy rats, the inputs from the medial and lateral EC (via temporoammonic and perforant inputs) evoked a firing of hippocampal neurons, which reflects an integrated representation of spatial and temporal information (O’Keefe and Nadel, 1978; Komorowski et al., 2009; Mankin et al., 2012; Kraus et al., 2013; Kitamura et al., 2014) as well as new experience (Frank et al., 2000; Wood et al., 2000). This neuronal coding is precisely organized within a time scale, which is controlled by ongoing oscillations, especially by the hippocampal theta and gamma rhythms (Bland and Oddie, 2001; Hasselmo et al., 2002; Huxter et al., 2008; Mizuseki et al., 2009; Easton et al., 2012; Buzsáki and Moser, 2013; Lisman and Jensen, 2013). A careful measurement of the proximodistal coherence of the theta activity in the dorsal hippocampus of normal and epileptic animals showed that healthy rats exhibited a stronger coordination between the temporoammonic and perforant entorhinal inputs near CA3 field (at proximal locations), while epileptic rats showed stronger coordination near subiculum (at distal locations; Laurent et al., 2015). This opposing trend in epileptic rats was associated with the connectivity constraint, which accompanies cell death in the hippocampus. Laurent et al. (2015) also discovered that the “appropriate timing between entorhinal inputs arriving over several theta cycles at the proximal and distal ends of the dorsal hippocampus was impaired in epileptic rats.” It is important that “the computational reconstruction of LFP signals predicted that timing variability has a major impact on repairing theta coherence.” Thus, the proximodistal organization of entorhinal inputs plays an important role in temporal lobe physiology, and this organization alters during TLE (Laurent et al., 2015).

#### Hippocampal–Medial Prefrontal Cortex Network

As was mentioned above, experiments with healthy animals showed that theta and gamma oscillations are usually present and work in synchrony in the hippocampus and medial prefrontal cortex (mPFC) during the performance of cognitive tasks (Tort et al., 2008; Benchenane et al., 2010). Hippocampal theta oscillations are normally coupled to mPFC theta waves (Benchenane et al., 2010) and modulate hippocampal and mPFC gamma oscillations during cognitive behavior (Jones and Wilson, 2005; Siapas et al., 2005; Tort et al., 2008). In a TLE model generated by perforant path stimulation, abnormal changes in the hippocampal−mPFC circuit were observed during the recording of mPFC and hippocampal LFPs in rats with spontaneous recurrent seizures (Broggini et al., 2016). Broggini et al. (2016) showed that recurrent seizures weaken hippocampal theta rhythm while the hippocampal and mPFC theta coherence increases during a period preceding the onset of seizures (Figure 1F). Simultaneously with the increase in theta synchrony a stronger coupling between hippocampal theta and mPFC gamma oscillations was observed (Figures 2E,F). Using the Granger causality, it was shown that the increase in hippocampus–mPFC synchrony in the preictal phase was provoked by hippocampal networks. The data indicate that the increase in hippocampal—mPFC coherence may predict the seizure onset (Broggini et al., 2016). Besides, the too strong coupling of hippocampal theta and mPFC gamma oscillations may induce abnormal plasticity in mPFC communications (Zheng and Zhang, 2015), which can be a reason of changes observed in mPFC cells (Tang and Loke, 2010).

#### Hippocampal–Septal Network

The registration of LFPs in the hippocampus and medial septal-diagonal band (MSDB) complex of rats and guinea pigs revealed that normally theta oscillations were relatively synchronous in these brain regions (Nerad and McNaughton, 2006; Astasheva and Kichigina, 2009; Kabanova et al., 2011). Usually theta power in the MSDB was smaller compared to that in the hippocampus, but the frequency of theta oscillations, although it did not coincide in these structures, did not differ significantly. The theta coherence between the hippocampus and MSDB was relatively high: a phase analysis revealed no clear unidirectional shifts (<10 ms) in the hippocampal and MSDB theta phases in healthy animals (Nerad and McNaughton, 2006; Kabanova, 2011; Kabanova et al., 2011). In chronic epileptic animals, a significant decrease of the theta power was revealed in the hippocampus (Arabadzisz et al., 2005; Colom et al., 2006; Dugladze et al., 2007; Astasheva and Kichigina, 2009; Marcelin et al., 2009) and MSDB (Sinelnikova, 2012). In addition, in a pilocarpine rat model of TLE, a dysfunctional and uncoupled septohippocampal network was revealed (García-Hernández et al., 2010). However, in the perforant path kindling model of TLE some increase in synchronization between hippocampus and MSDB within the theta band was observed in waking guinea pigs during epileptogenesis (Figure 1G; Kabanova et al., 2011). Besides, in this model of TLE, a dramatic increase of the theta oscillations simultaneously in the rabbit hippocampus and MSDB before (within 20 s) the seizures was observed (Kitchigina and Butuzova, 2009). This phenomenon reminds the events in the hippocampal–mPFC network over time prior to seizure onset in rats in the same model of TLE (Broggini et al., 2016). Interestingly, in a perforant path kindling model of TLE in guinea pigs, the interactions between the hippocampus and MSDB changed for opposite during epileptogenesis: at the beginning of kindling, the MSDB was ahead in the theta phase, but after formation of the pathological focus, MSDB lagged the hippocampus (Kabanova et al., 2011). In addition, the relationships between rhythmic bursts of septal neurons and the phases of the hippocampal theta waves during spontaneous seizures in rabbits with TLE model could reverse to almost opposite comparative to interictal ones (Kitchigina and Butuzova, 2009); i.e., these relationships were not constants.

It was shown in earlier experiments that the natural theta rhythm evoked, e.g., by sensory stimuli prevents seizure onset under the influence of epileptogenic factors (Miller et al., 1994; Colom et al., 2006; Kitchigina and Butuzova, 2009). At the same time, excessive theta synchrony leads to the generation of epileptiform activity (Kitchigina and Butuzova, 2009). Thus, for the prevention of seizure development, a strong control of the level and pattern of the hippocampus—MSDB theta synchronization is necessary.

### Alterations in the Rhythm Coherence in Alzheimer’s Disease and in the AD Models

#### Disturbances of Theta and Gamma Rhythms in Brain With AD Pathology

AD is a progressive neurodegenerative disease associated with an irreversible deterioration of cognitive functions, especially memory. Although the etiology of AD remains unknown and now there is no reliable treatment, a consensus has emerged early in this century on the amyloid hypothesis (Selkoe, 2000; Palop and Mucke, 2010), which posits that the amyloid β (Aβ) peptide, a major constituent of amyloid plaques, is mostly responsible for the alteration of cognitive functions (Francis et al., 2010; Palop and Mucke, 2010). In the last years, however, this hypothesis was challenged: a potential role of an impairment of metabolism of amyloid precursor protein (APP) and its progress through tau pathology were considered in the etiology of AD (for review, see Kametani and Hasegawa, 2018). Moreover, the recent data of experiments with wild-type and APP/PS1 transgenic mice indicate that amyloid plaques can possess capacity for binding additional Aβ (Gureviciene et al., 2017).

Various forms of memory are disturbed in AD (Didic et al., 2011). It has been assumed that navigation deficits can help to separate individuals at higher risk of developing AD from patients with other neurodegenerative diseases (Lithfous et al., 2013). As it was revealed in some works, AD patients, as opposed to healthy age-matched control subjects, exhibit an increase in the relative power of slow oscillations (in particular, theta rhythm) and a decrease in the relative power of fast oscillations (gamma rhythm; Adler et al., 2003; Herrmann and Demiralp, 2005; van der Hiele et al., 2007; Czigler et al., 2008; Moretti et al., 2010). On the contrary, in other works, an increased gamma rhythm power and the lack of theta increase in AD patients were reported (Caravaglios et al., 2010; Wang et al., 2017). Some authors noted that changes in EEG of resting AD patients might not be specific, and various types of dementia can also exhibit similar network disturbances (Herrmann and Demiralp, 2005). Besides, contrary to the data on AD patients, a decrease of both theta and gamma bands was revealed in Tg5xFAD mice, a transgenic mouse model of AD; in this case, the decrease preceded alterations in learning performances in spatial task (Schneider et al., 2014). In addition, transgenic APP23 mice, another mouse model of AD, demonstrated the compromised spectral contributions of hippocampal theta and gamma oscillations, compared to non-transgenic controls: a markedly lower spectral power of theta oscillations (~10 Hz) and a higher power of gamma oscillations (25–50 Hz; Ittner et al., 2014), changes opposite to those in AD patients. Hence, a decrease or an increase in theta and gamma oscillations power *per se* may not be specific for this pathology (Herrmann and Demiralp, 2005).

#### Alterations in Theta–Gamma Coherence Are Indicative for Brain With AD Pathology

Probably, most convincing evidence of rhythm disturbances in a pathological AD brain is alterations in the theta–gamma CFC. Thus, in humans with AD, an enhanced CFC between the gamma and low-frequency bands (in particular, theta) compared to healthy control was revealed (Wang et al., 2017). During performance of working memory tasks, evidence for a relationship between altered theta-gamma coupling and working memory deficits in individuals with AD was obtained (Goodman et al., 2018).

**Figure 3:**
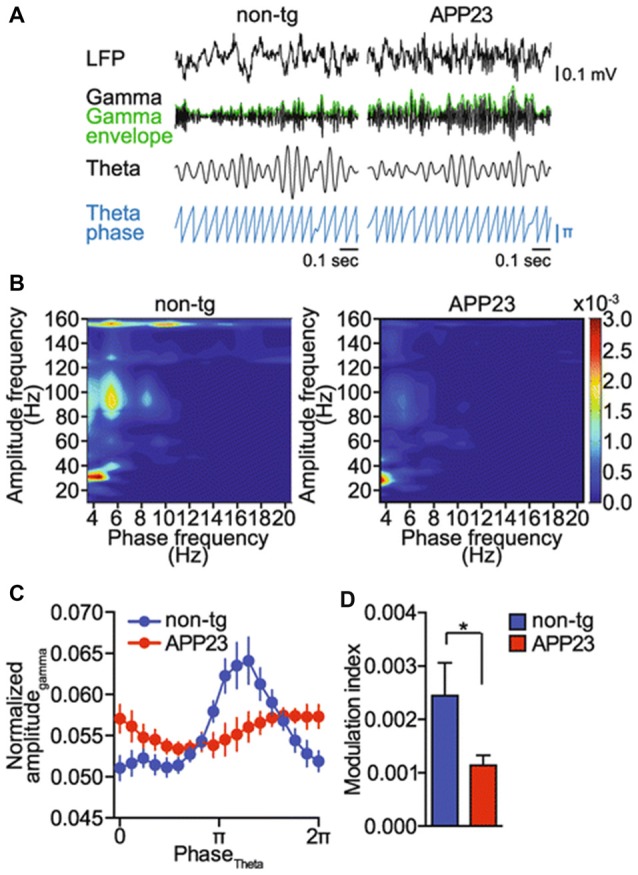
Gamma amplitude modulation by theta phase is impaired in a mouse model of Alzheimer’s disease (AD; amyloid precursor protein 23, APP23 mice). **(A)** Raw EEG (LFP), band pass filtered signals for theta (4–12 Hz) and gamma (25–100 Hz) oscillations, gamma amplitude envelope (green) and theta phase in APP23 and non-transgenic (non-tg) mice (blue). Representative signals from five animals per genotype are shown. **(B)** Representative phase–amplitude comodulograms computed for hippocampal LFPs recorded in non-tg and APP23 mice. **(C)** Phase–amplitude plot computed for hippocampal LFPs recorded in non-tg and APP23 mice (means ± SEM). **(D)** MI computed for the phase–amplitude distributions shown in **(C)**; *p < 0.05. Adapted from Ittner et al. (2014; Open Access).

In the AD model (adult APP23 transgenic free-roaming mice), an impairment of cross-frequency gamma amplitude modulation by hippocampal theta rhythm was observed (Ittner et al., 2014; Figure 3). It is important that these changes were observed before the onset of Aβ plaque pathology. Moreover, it was shown on TgCRND8 mice that a significant proportion of 1-month-old animals exhibited marked alterations in the theta–gamma coupling in the output region of the hippocampus, the subiculum. This uncoupling of rhythms arises before any histopathological abnormalities such as the presence of amyloid plaques (Goutagny et al., 2013). In addition, it was shown that 1-month-old TgCRND8 mice expressed extremely low levels of Aβ compared to controls. Goutagny et al. (2013) suggested that in animals (TgCRND8 mice) disturbed theta–gamma CFC in the subiculum may be the earliest detectable AD-related biomarker. This is in contrast with the existing hypothesis, which states that the beginning of hippocampal network alterations and memory deficits in animal models of AD are caused by the overproduction of soluble Aβ (Francis et al., 2010; Palop and Mucke, 2010; Scott et al., 2012).

Interestingly, though APP is supposed to be critically involved in the pathophysiology of AD, APP-deficient mice exhibit cognitive deficits (Seabrook et al., 1999; Senechal et al., 2008); this confirms that APP plays an important role in the functioning of neurons in the healthy brain. Recently, strongly diminished theta–gamma coupling in LFPs from the dorsal hippocampus and parietal cortex was revealed in APP knockout mice. Besides, cross-regional hippocampal–prefrontal CFC was largely disrupted in these knockout mice (Zhang et al., 2016). This effect may be of importance for the origination of cognitive deficits in APP-deficient animals. Thus, APP is important for the interaction of rhythms of different frequencies. The facts mentioned above possibly indicate, that very thin frontier between functioning of APP in the healthy and pathological brains exists.

**Figure 4:**
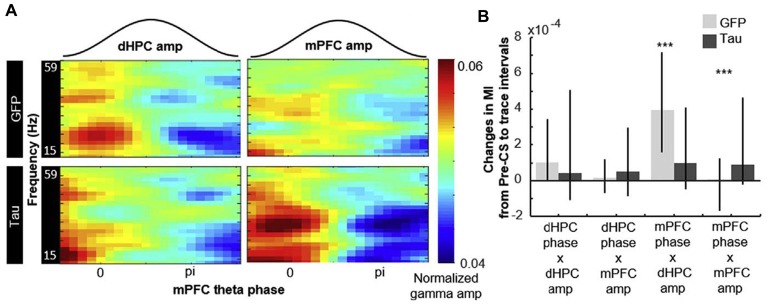
Entorhinal tau overexpression affects phase–amplitude hippocampal—prefrontal CFC. The degree of modulation of gamma amplitude by mPFC theta phase quantified with the MI. **(A)** Representative examples of normalized hippocampal (to the left) and prefrontal (to the right) gamma amplitude aligned to concurrent prefrontal theta phase. **(B)** Changes in the MI before and after the CS (median ± 25th and 75th percentile). Compared against GFP-expressing rats (light gray), the change in MI of prefrontal theta-hippocampal gamma coupling was smaller while that of prefrontal theta-prefrontal gamma coupling was larger in tau-expressing rats (dark gray); ***p < 0.001. Abbreviations: CS, conditioned stimulus; GFP, green fluorescent protein. Adapted with permission from Tanninen et al. (2017).

Quite recently, it has been tested whether a preclinical AD pathologic feature, tau aggregation in the EC, can disrupt the coordination of LFPs between its two efferent regions, the hippocampus and prelimbic mPFC (Figure 4; Tanninen et al., 2017). Tanninen and colleagues revealed strengthened phase–phase and amplitude–amplitude couplings of theta and gamma oscillations in these two regions during associative learning in healthy rats (the rats underwent trace eyeblink conditioning and were learned to associate two stimuli separated by a short time interval). In tau-expressing rats, the hippocampus and PFC showed a significant attenuation of stimulus-evoked theta oscillations. In addition, despite normal memory acquisition, the learning-related oscillatory coupling between the hippocampus and the PFC in these rats was diminished; at the same time, the entorhinal tau overexpression enhanced the stimulus-evoked theta–gamma phase–amplitude coupling within the mPFC (Figure 4). The authors suggested that the tau aggregation in the EC caused aberrant long-range circuit activity during associative learning, indicating the disturbances in neural oscillations of preclinical AD stages (Tanninen et al., 2017).

The tau aggregation has been also described in epileptic patients and animals with marked cognitive disturbances (Thom et al., 2011; Tai et al., 2017, 2018). Though the data about rhythmic abnormalities are absent in the works mentioned, these disorder can be expected to occur.

## Electro-Clinical Data for Patients With “Epileptic Prodromal AD”

A similarity in the alterations of oscillatory activity in the AD/TLE brain (in particular, disturbances in theta-gamma coherence in hippocampal-cortical networks) suggests that these diseases have some common properties and, probably, at least partially similar mechanisms of their development.

The potential relation between TLE and AD has been supported by experimental and clinical data. Thus, aging is a common and well-established risk factor for epilepsy and AD (Armon et al., 2000; Maguire and Frith, 2003; Amatniek et al., 2006; Bernardi et al., 2010; Born et al., 2014; Chan et al., 2015). Besides, AD may be an important cause of epileptic disorders, as shown in elderly humans (Armon et al., 2000; Bird et al., 2005; Bernardi et al., 2010; Palop and Mucke, 2016) and in animals with AD models (Bezzina et al., 2015; Chan et al., 2015). Patients with AD have a 5- to 10-fold increased risk of the development of seizures or other forms of epileptiform activity (Amatniek et al., 2006). Although seizure pathology was previously believed to be secondary to AD, it was found that neuronal activity can regulate regional vulnerability to Aβ (Palop et al., 2007; Palop and Mucke, 2010; Bero et al., 2011); in particular, enhanced neuronal excitability can increase Aβ generation (Cirrito et al., 2008). Moreover, disturbed activity may contribute to the development of cognitive violations: epileptiform and rhythmic abnormalities in the temporal regions (in particular in the hippocampus) can cause amnestic disorders, which were reduced by antiepileptic drug treatment (Gallassi, 2006; Bakker et al., 2012). In patients with seizures in combination with AD, a case series from California with so called “vu/déjà vu” phenomena was described (Vossel et al., 2013), while another series from France (Cretin et al., 2016) had some cases that were termed “epileptic prodromal AD.” The authors believed that there is an epileptic version of AD, which usually starts with seizures as an initial symptom followed by cognitive deficit. Similar signs of cognitive and behavioral impairments in TLE and AD have been recently described by Chin and Scharfman (2013).

Many clinical evidence indicates an increased comorbidity of seizure pathology in AD: it is becoming clear that AD is associated with neuronal hyperexcitability as well as network hypersynchronicity, which is the main reasons of epilepsy development (Eichler and Meier, 2008; Noebels, 2011; Saito et al., 2012; Varga et al., 2014). Indeed, epileptic prodromal AD patients suffer from seizures sometimes even before developing clear cognitive disorders. The epileptiform activity may manifest itself in the early stages of AD more often than was previously proposed. Thus, seizures in patients with AD and amnestic mild cognitive impairment are associated with an earlier appearance of cognitive decline (Amatniek et al., 2006; Scarmeas et al., 2009; Irizarry et al., 2012; Vossel et al., 2013). In the study of Sarkis and colleagues, the authors describe patients with recurrent medically refractory epileptic auras, which ultimately lead to the disease diagnosed as AD (Sarkis et al., 2017).

At the same time, neurodegenerative processes peculiar to dementia can play a central role in the development of epilepsy in the patients predisposed to cognitive deficit. Adult-onset epilepsy of unknown cause could thus represent a risk factor for the ongoing neurodegenerative damage, even when epileptic manifestations and clinically recognized dementia are separated by long time (Cretin et al., 2016; Sarkis et al., 2017).

The facts of cognitive impairment in animals with epileptiform activity were derived from different studies devoted to the role of the tau peptide (Roberson et al., 2011) or APP overexpression in mouse models of AD (Born et al., 2014). Genetic suppression of the APP level resulted in a normalization of EEG activity (Born et al., 2014) as well as the tau reduction was beneficial for animals in multiple models of AD (Roberson et al., 2007, 2011; Gómez de Barreda et al., 2010; Ittner et al., 2010). Unfortunately, this is only true in animal models of the disease, not in AD patients.

In the hippocampus, one of the main foci of cell death in TLE and AD brains, the network hypersynchronicity and epileptiform activity can be the result of formation of extensive aberrant neuronal connections. This aberrant remodeling was revealed in epileptic rats and in APP transgenic mice (Harris et al., 2003; Palop et al., 2007; Minkeviciene et al., 2009; Palop and Mucke, 2010; Vogt et al., 2011). The aberrant reconstruction can be a cause of alterations in the oscillatory activity and rhythm coherence in brains with TLE and AD pathologies.

## Conclusion

It is known that the main problem in diagnosis of neurodegenerative diseases is the detection of neuronal abnormalities at early stages of their development. At present significant achievements have been made in the development of methods for the detection of some biomarkers of AD and TLE, including cerebrospinal fluid and plasma measurements and glucose positron emission tomography (Shiihara et al., 2006; Scholl-Bürgi et al., 2008; Mattsson et al., 2009; Shaw et al., 2009; Visser et al., 2009; Blennow et al., 2007; Jack et al., 2011; van Karnebeek et al., 2012). However, there is an urgent need for biomarkers that would reflect changes in brain functioning within few milliseconds to obtain information about the progressing cognitive deficiency (Yener and Basar, 2013; Wang and Meng, 2016). The application of magnetoencephalography in combination with the coherent analysis, in particular during cognitive loading, is a promising approach to early diagnosis of these diseases. Thus, the specific disturbances in interactions of theta–gamma oscillations in hippocampal, hippocampal–entorhinal, hippocampal–prefrontal and hippocampal–septal networks were revealed in the epileptic brain. In the AD models, marked changes were observed in the theta–gamma coupling in the subiculum, an output region of the hippocampus. In addition, a decreased theta–gamma coupling between the hippocampus and the parietal cortex as well as between the hippocampus and the PFC was also shown.

At present, the methods for detection of theta–gamma coherence during cognitive loading are still not absolutely perfect. Thus, changes in theta–gamma coupling may simply reflect memory-related increases in gamma power and phase synchrony (Montgomery and Buzsáki, 2007). Novel approaches (in particular, optogenetics) should allow one to alter the relationship between gamma power and theta phase without affecting theta/gamma rhythms themselves (Colgin, 2015). This manipulation would enable one to directly determine how coupling between theta and gamma oscillations affects neuronal activity and memory operations in the brain. New approaches would help to elaborate precise early biomarkers for the diagnosis of AD and TLE. The advances of coherence methods in the detection of rhythm violation will help to deepen our understanding of the mechanisms of disturbances in theta–gamma relationship in the AD/TLE brain. Possibly, in future, specific disturbances in theta–gamma coherence will serve as markers of particular cell damage and will allow one to direct therapeutic influences to certain neural loci at early stages of the development of the disease.

## Author Contributions

The author confirms being the sole contributor of this work and approved it for publication.

## Conflict of Interest Statement

The author declares that the research was conducted in the absence of any commercial or financial relationships that could be construed as a potential conflict of interest.
